# Effect of Tocilizumab in Reducing the Mortality Rate in COVID-19 Patients: A Systematic Review with Meta-Analysis

**DOI:** 10.3390/jpm11070628

**Published:** 2021-07-01

**Authors:** Valeria Conti, Graziamaria Corbi, Carmine Sellitto, Francesco Sabbatino, Chiara Maci, Nicola Bertini, Emanuela De Bellis, Antonio Iuliano, Sergio Davinelli, Pasquale Pagliano, Amelia Filippelli

**Affiliations:** 1 Department of Medicine, Surgery and Dentistry “Scuola Medica Salernitana”, University of Salerno, 84081 Salerno, Italy; vconti@unisa.it (V.C.); csellitto@unisa.it (C.S.); fsabbatino@unisa.it (F.S.); chiaramaci7@gmail.com (C.M.); nicobertini90@gmail.com (N.B.); e.debellis93@gmail.com (E.D.B.); a.iuliano@hotmail.it (A.I.); ppagliano@unisa.it (P.P.); afilippelli@unisa.it (A.F.); 2 Clinical Pharmacology and Pharmacogenetics Unit, University Hospital “San Giovanni di Dio e Ruggi D’Aragona”, 84125 Salerno, Italy; 3 Department of Medicine and Health Sciences, University of Molise, 86100 Campobasso, Italy; sergio.davinelli@unimol.it; 4 Italian Society of Gerontology and Geriatrics (SIGG), Via G.C. Vanini, 5, 50129 Firenze, Italy; 5 Oncology Unit, University Hospital San Giovanni di Dio e Ruggi D’Aragona, 84125 Salerno, Italy; 6 Infectious Diseases Unit, Hospital San Giovanni di Dio e Ruggi D’Aragona, 84125 Salerno, Italy

**Keywords:** tocilizumab, standard therapy, COVID-19 mortality, critical and severe patients, anti-inflammatory drugs

## Abstract

Data supporting the use of Tocilizumab (TCZ) in COVID-19 are contrasting and inconclusive. This meta-analysis aimed to assess TCZ effectiveness in reducing the mortality rate in COVID-19 patients. PubMed, Scopus, Embase, Cochrane, WILEY, and ClinicalTrials.gov were searched to evaluate observational studies and RCTs. The outcome was the mortality rate. Forty observational studies and seven RCTs, involving 9640 and 5556 subjects treated with Standard Therapy (ST) + TCZ or ST alone, respectively, were included. In patients treated with ST+TCZ, a higher survival (Log odds ratio = −0.41; 95% CI: −0.68 −0.14; *p* < 0.001) was found. Subgroups analyses were performed to better identify the possible interference of some parameters in modifying the efficacy of TCZ therapy on COVID-19 mortality. Separating observational from RCTs, no statistically significant (*p* = 0.70) TCZ-related reduction of mortality regarding RCTs was found, while a significant reduction (Log odds ratio = −0.52; 95% CI: −0.82 −0.22, *p* < 0.001) was achieved regarding the observational studies. Stratifying for the use of Invasive Mechanic Ventilation (IMV), a higher survival was found in patients treated with TCZ in the No-IMV and IMV groups (both *p* < 0.001), but not in the No-IMV/IMV group. Meta-regression analyses were also performed. The meta-analysis of observational studies reveals that TCZ is associated with reducing the mortality rate in both severe and critically ill patients. Although the largest RCT, RECOVERY, is in line with this result, the meta-analysis of RCTs failed to found any difference between ST + TCZ and ST. It is crucial to personalize the therapy considering the patients’ characteristics.

## 1. Introduction

The Coronavirus disease 2019 (COVID-19) pandemic, caused by Severe Acute Respiratory Syndrome Coronavirus 2 (SARS-CoV-2), represents a very difficult challenge. Both repositioned and experimental drugs have been used to treat infected patients, often without evidence of efficacy [1,2]. Clinical presentation of COVID-19 ranges from asymptomatic to severe cases that finally evolve toward refractory hypoxemia [3]. Thus far, patient management essentially consists of steroids and heparin administration coupled with non-invasive or invasive respiratory support. As a result, COVID-19 results in a global fatality rate exceeding 2% [4,5,6], highlighting the urgent need to identify effective treatments to prevent morbidity and mortality from this infection.

Mortality by COVID-19 is caused by an Acute Respiratory Distress Syndrome (ARDS) or multiorgan failure [7]. Several pathophysiological mechanisms have been proposed to explain such outcomes [8]. Most of the studies demonstrated that patients with progressively severe symptoms present the so-called COVID-19-associated Cytokine Storm Syndrome (CSS), which consists of an inadequate systemic inflammatory response of the host immune system to viral infection [9]. CSS is characterized by a rapid and significant increase in the serum level of pro-inflammatory cytokines such as IL-1-β, IL6, IFNγ, MCP, and TNFα. IL-6 plays a key role in the clinical manifestations of COVID-19 disease and represents a predictive marker of fatal outcome [10,11]. A similar syndrome complication by aberrant IL-6 release is well described in leukemic patients treated with chimeric antigen receptor T-cell (CAR-T) therapy. As a result, several drugs targeting the IL-6 pathway have been attempted, including tocilizumab (TCZ) [12]. TCZ is an anti-IL-6 receptor monoclonal antibody currently approved for the treatment of several forms of arthritis as well as for cytokine release syndrome related to CAR-T therapy [13,14]. Preliminary clinical data and then several observational retrospective studies have shown an improvement in pneumonia and associated symptoms in COVID-19 patients treated with TCZ [2]. However, contrasting data have emerged on the routine use of this drug [15].

This meta-analysis aimed to review and assess the effectiveness of TCZ in reducing the mortality rate in COVID-19 patients.

## 2. Materials and Methods

The study follows the recommendations of MOOSE guidelines for Meta-Analysis and Systematic Reviews of Observational Studies [16]. The PRISMA statement of reporting systematic review and meta-analysis [17] was applied. The study was registered on PROSPERO (CRD42021223124).

### 2.1. Search Strategy

A comprehensive systematic literature search was performed using both controlled vocabulary and free text terms. The following Medical Subject Heading (MESH) terms were used, by using Boolean operators “AND” and “OR”: COVID-19; antibodies, monoclonal; tocilizumab; immunosuppressive agent; mortality; survival; SARS-CoV-2. The databases PubMed, Scopus, Embase, Cochrane, WILEY, and ClinicalTrials.gov were searched from inception up to May 2021 to find studies reporting the relationship between TCZ administration and the mortality rate in patients with COVID-19. 

### 2.2. Study Selection, Data Extraction and Quality Assessment

Our research was limited to studies involving humans. Observational studies and Randomized Clinical trials (RCTs) were eligible, while case reports, editorials, reviews, and abstracts were excluded. Only studies that clearly assessed the effect of TCZ on mortality in patients with a diagnosis of COVID-19 and that compared the mortality rate between patients treated with TCZ added to standard therapy (ST) and those treated with ST alone were included.

To focus on the research question, a P.I.C.O. model was used (Table 1). 

The selection criteria were developed by two reviewers (G.C., V.C.). Two authors (C.M., A.I.) independently screened the titles and abstracts of all the articles were retrieved to identify those eligible according to the inclusion criteria. Full texts of the selected studies were then screened for eligibility by 2 independent reviewers (N.B. and F.S.). Potential disagreement about the eligibility of a study was resolved by a third author (S.D.). Data from all the selected articles were extracted by two authors (C.S. and E.D.B.) and checked by two other authors (G.C. and V.C.). The following information was recorded: author’s name, publication year, study country, study design, sample size, participant characteristics, health status, standard therapy for COVID-19, intervention (treatment with or without TCZ), and mortality. The quality of the studies was assessed using the Newcastle–Ottawa Score for the observational studies [18] and the Cochrane Risk of Bias tool for the RCTs [19,20]. A GRADE analysis for both the observational studies and RTCs was also performed (Suppl Figures S1B and S2B).

Two reviewers independently assessed the study quality and risk of bias (G.C. and S.D.), and discrepancies were resolved by discussion with a third reviewer (V.C.).

### 2.3. Statistical Analysis

Participants who were treated with ST + TCZ were recorded as the Tocilizumab group, while those who were treated with ST alone were recorded as the ST group. Data were expressed as the number of subjects who died or survived in each group. Random-effects models were prespecified a priori, given the heterogeneity across settings, participants, and sample size [21]. 

Data were summarized across treatment arms using the Mantel–Haenszel odds ratio (OR). A two-sided probability *p*-value < 0.05 was considered statistically significant. Statistical heterogeneity was tested using the Cochran Q statistic and quantified by the I2 value [22] as follows: I2 < 25% (very low), 25 to <50% (low), 50 to <75% (moderate), and ≥75% (large) [23,24]. Given significant heterogeneity, sensitivity analyses were performed by removing a single study at a time to determine how robust the findings were. When heterogeneity was small or absent in a random-effects model, a fixed-effects model was used to confirm the consistency. Forest plots were used to visualize the results. 

Given significant heterogeneity in the observational studies, a meta-regression analysis was performed using differences in mean age and percentage of female population as moderators. Moreover, subgroup analyses were conducted considering step-by-step the use of Invasive Mechanical Ventilation (IMV), the use of steroids, the type of study (Perspective/Retrospective), and the TCZ dose (one dose/more than one dose). To also evaluate the effects of these factors, other meta-regressions were performed based on the available data (Table S1).

Funnel plots were used initially to evaluate visually publication bias, while Egger’s regression test was used to inferentially evaluate publication bias [25]. Statistical analysis was performed using Stata 16 version.

## 3. Results

### 3.1. Search Results

**Figure 1:**
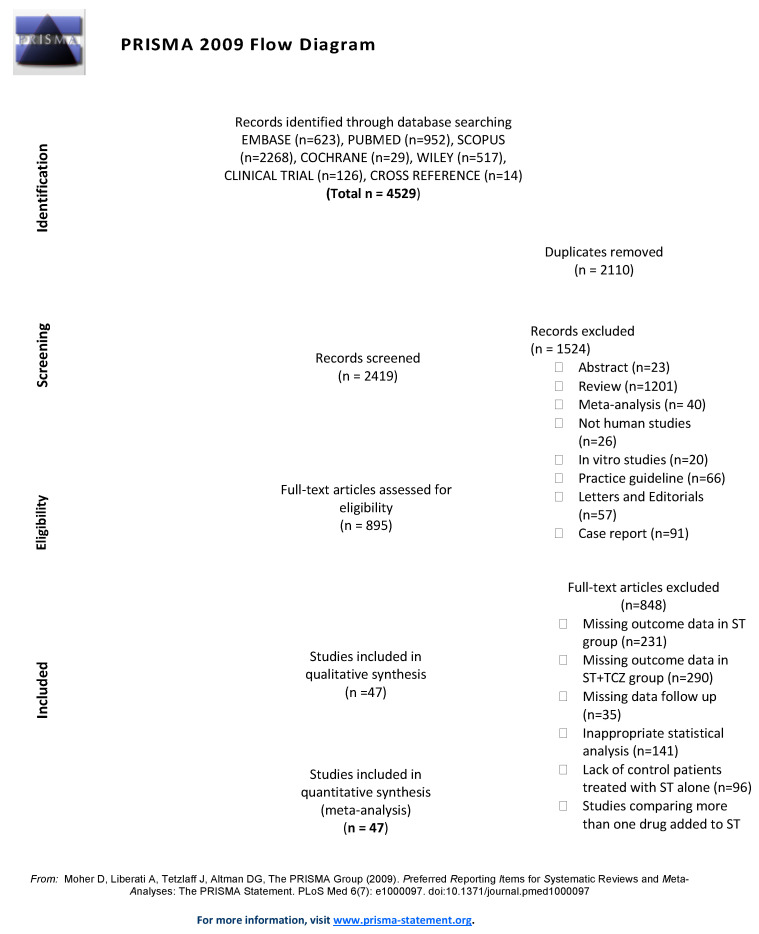
The PRISMA algorithm of the study.

The PRISMA algorithm shows the flow of records through the review (Figure 1). 

A total of 47 studies, 40 observational studies [26,27,28,29,30,31,32,33,34,35,36,37,38,39,40,41,42,43,44,45,46,47,48,49,50,51,52,53,54,55,56,57,58,59,60,61,62,63,64,65] reported in Table 2 and 7 RCTs [66,67,68,69,70,71,72] reported in Table 3, were included in the final analysis.

### 3.2. Study Characteristics

Table 2 summarizes the characteristics of the included observational studies addressing the effect of TCZ therapy on the mortality of COVID-19 patients. Thirty-one were single-center and nine were multicenter studies. A total of 9640 patients were involved: 3085 and 6355 patients were treated with TCZ + ST and ST alone, respectively. The mean age of all participants was 64.44 ± 13.89 years (range 53–78 years). 

The ST most frequently administered consisted of hydroxychloroquine (HCQ) (400 mg/die) and Lopinavir/ritonavir (Lop/r, 400/100 bid) or darunavir/cobicistat (800/150 mg). Patel et al. [52] reported the use of remdesivir or Lop/r, while Gokhale et al. [38] described the administration of oseltamivir. Twenty-two studies reported the use of steroids as part of ST, while 15 did not. No information on the administration of steroids was reported in three studies. 

As shown in Table 2, 17 studies reported data on patients who were not receiving IMV (No-IMV) at the initiation of TCZ, 18 analyzed a mixed population including both No-IMV and IMV patients, and 5 included patients who were using IMV at the initiation of TCZ (Table 2).

Two studies (Pan-Li and Rojas-Marte) included information on subjects that did or did not use IMV; therefore, they were introduced separately in the groups.

Table 3 summarizes the characteristics of the included RCTs addressing the effect of TCZ therapy on the mortality of COVID-19 patients. All the RCTs were multicenter studies. A total of 5556 patients were involved: 2914 were treated with TCZ+ST and 2642 with ST alone. The mean age of all the participants was 60.10 ± 14.05 years. In three RCTs (COVACTA, RECOVERY, and TOCIBRAS) a mixed use of IMV/No-IMV was reported, while in the remaining four RCTs, No-IMV support was referred.

All the RCTs included the use of steroids in the ST. Only one RCT (RCT-TCZ-COVID-19) reported the use of more than one TCZ dose, while the remaining six all reported the use of only one TCZ dose.

### 3.3. Quality Assessment

In all the studies, both the cases and controls were hospitalized for COVID-19 infection. None of the studies indicated whether the participants were first-time COVID-19 patients or not. The quality score for the observational studies, achieved by using the Newcastle–Ottawa Scale, ranged from 7 to 9 with a mean score of 8.2 (Suppl Figure S2A). The GRADE analysis revealed a good quality for the RCTs and a moderate quality for the observational studies included in the meta-analysis (Suppl Figures S1B and S2B). 

### 3.4. Meta-Analysis of the TCZ Therapy on Mortality

Overall, we meta-analyzed 40 observational studies involving 3085 subjects treated with ST+TCZ and 6355 patients with ST alone, and 7 RCTs including 2914 subjects treated with ST+TCZ and 2642 patients with ST alone. Therefore, a total population of 14,996 subjects was analyzed. Meta-analysis showed a statistically significant higher survival in patients treated with ST+TCZ (Log odds ratio = −0.41; 95% CI: −0.68 −0.14; *p* < 0.001) as compared to subjects treated with ST alone.

**Figure 2:**
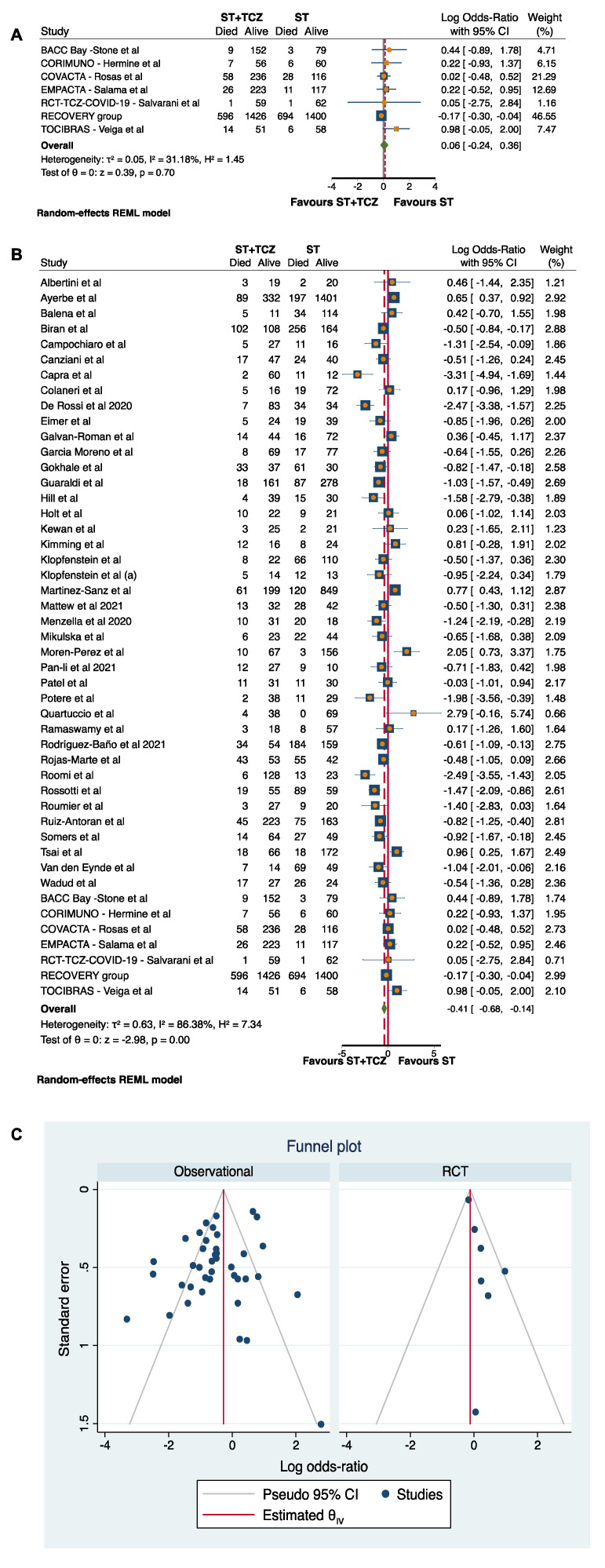
Forest plot in (**A**) RCTs and (**B**) observational studies. (**C**) Funnel plot of the subgroup analysis by RCTs and observational studies.

Due to the high heterogeneity among the studies (τ^2^ = 0.63, I^2^ = 86.38%, H^2^ =7.34), a subgroup analysis was carried out by discriminating between RCTs and observational studies (Figure 2). After this subdivision, the RCTs did not achieve any conclusive result (Log odds ratio = 0.06; 95% CI: −0.24 0.36; *p* = 0.70) (Figure 2A), while the 40 observational studies showed a statistically significant higher survival in patients treated with ST+TCZ (Log odds ratio = −0.52; 95% CI: −0.82 −0.22; *p* < 0.001) as compared to subjects treated with ST alone (Figure 2B). In Figure 2C, the funnel plot is shown. 

### 3.5. Sensitivity and Subgroup Analyses in RCTs

**Figure 3:**
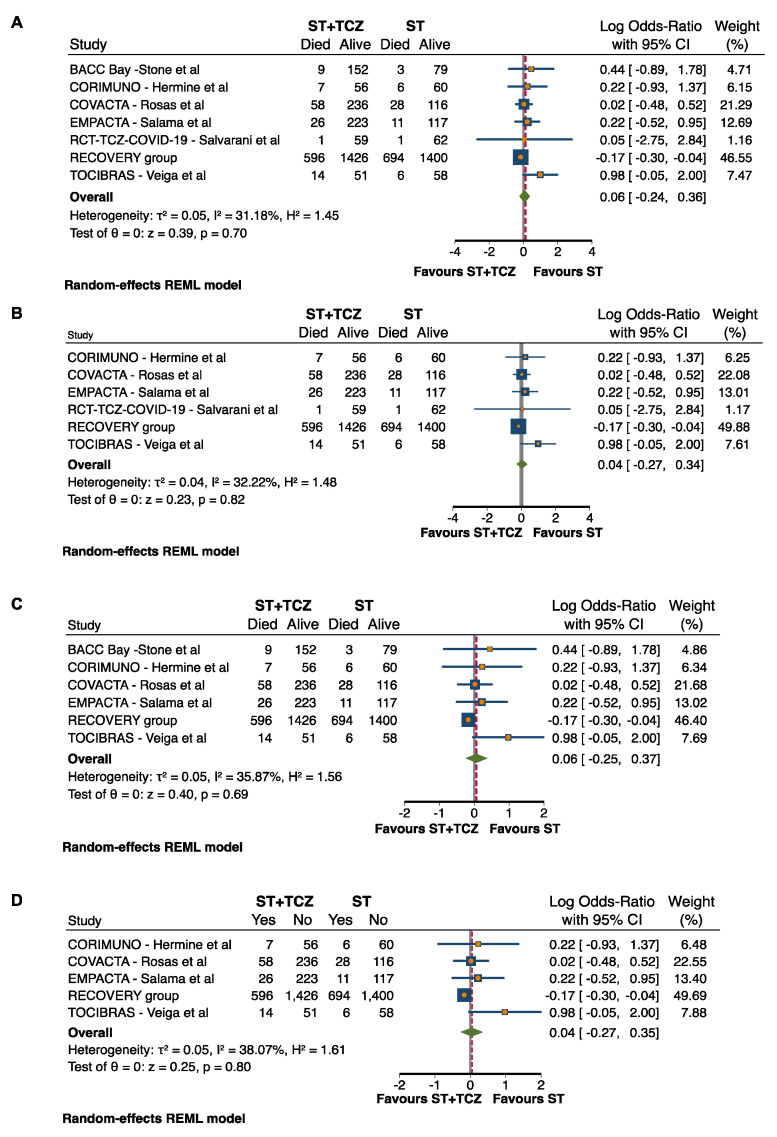
Forest plot in (**A**) all RCTs, after exclusion of RCTs (**B**) with weak allocation concealment, (**C**) with weak blinding, and (**D**) with both weak allocation concealment and blinding.

Based on the publication bias results, a sensitivity analysis was performed, excluding the step-by-step RCTs with weak allocation concealment (BACC) and weak blinding (RCT-TCZ-COVID-19) that represented the causes of risk of bias (Suppl Figure S1). In both cases, after the sensitivity analysis, the RCTs did not achieve any conclusive result (after BACC exclusion: Log odds ratio = 0.04; 95% CI: −0.27 0.34; *p* = 0.82; after RCT-TCZ-COVID-19 exclusion: Log odds ratio = 0.06; 95% CI: −0.25 0.37; *p* = 0.69) (Figure 3B,C). Additionally, when both the studies were excluded at the same time, the findings did not change (Log odds ratio = 0.04; 95% CI: −0.27 0.35; *p* = 0.80) (Figure 3D).

**Figure 4:**
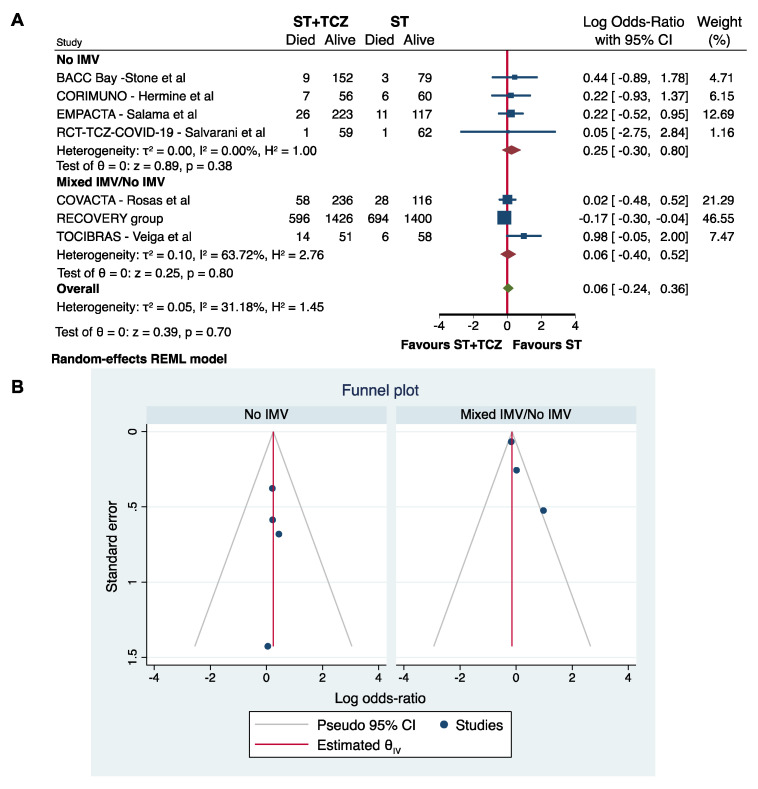
(**A**) Forest plot and (**B**) funnel plot of the subgroup analysis on the observational studies stratified by IMV use.

Although only a low–moderate heterogeneity characterized the RCTs, a subgroup analysis was performed using the IMV at the initiation of TCZ treatment as a stratifying variable. As previously, the RCTs did not achieve any conclusive results on the efficacy of TCZ to reduce COVID-19 mortality (No IMV: Log odds ratio = 0.25; 95% CI: −0.30 0.80; *p* = 0.38; Mixed IMV/No IMV: Log odds ratio = 0.06; 95% CI: −0.40 0.52; *p* = 0.80) (Figure 4A,B).

### 3.6. Sensitivity and Subgroup Analyses in the Observational Studies

**Figure 5:**
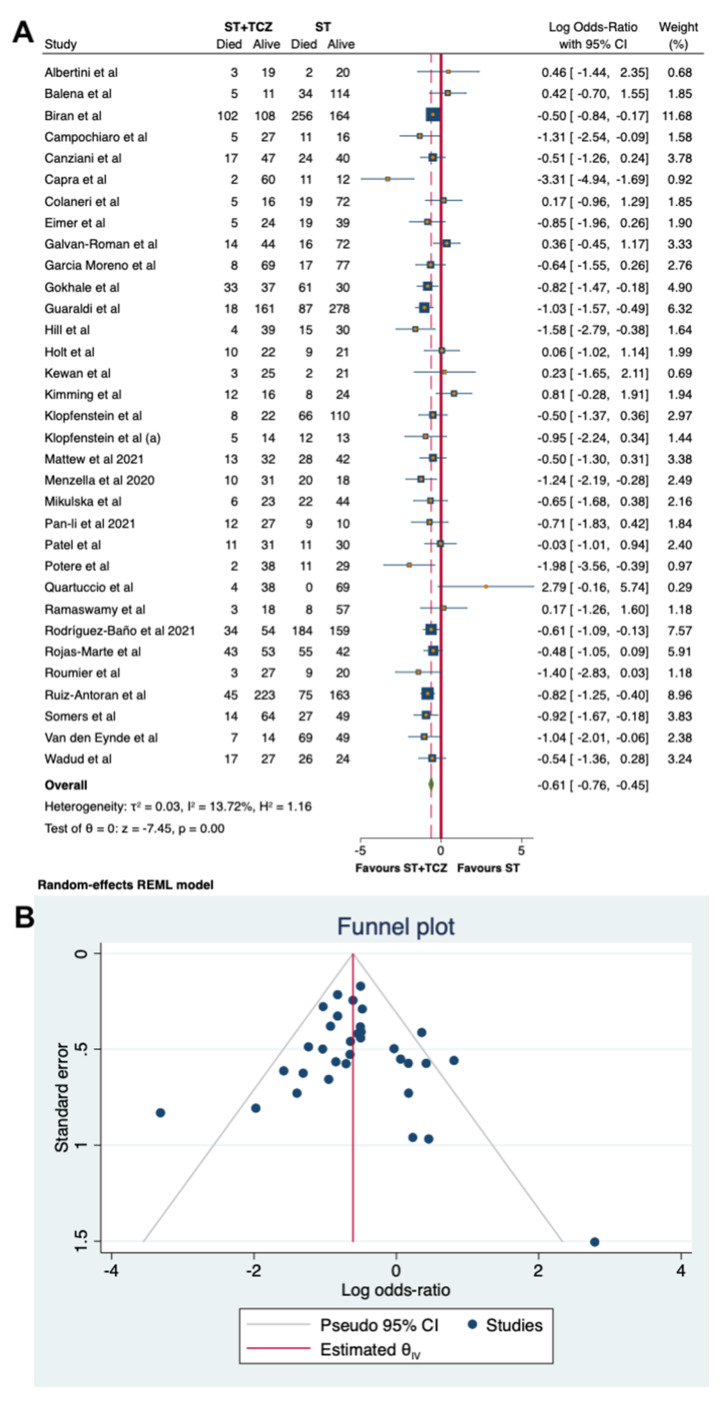
(**A**) Forest plot of the sensitivity analysis on observational studies by random-effects model. (**B**) Funnel plot of the sensitivity analysis on the observational studies.

Regarding the observational studies, because of the high heterogeneity, a sensitivity analysis was carried out, leading to the removal of seven studies. By using a random-effects model on the 33 remaining studies, involving 1945 patients with ST + TCZ and 3187 with ST alone, with a total number of subjects of 5326, a low heterogeneity was found (τ^2^ = 0.03, I^2^ = 13.72%, H^2^ = 1.16), confirming the findings obtained from the primary analysis (Log odds ratio = −0.61; 95% CI: −0.76 −0.45; *p* < 0.001, Figure 5A). 

The results were further confirmed by the sensitivity analysis performed with a fixed-effects Mantel–Haenszel model: the positive effects of TCZ therapy on mortality remained significant (Log odds ratio = −0.62; 95% CI: −0.75 −0.48; *p* < 0.001, Suppl Figure S2). A low heterogeneity has also been confirmed (I^2^ = 41.40%, H^2^ = 1.71). 

Figure 5B and Suppl Figure S2B show the funnel plots for the sensitivity analyses. The Egger’s test performed in both cases (overall studies *p* = 0.9375 and sensitivity of observational studies *p* = 0.7268, respectively) indicates no evidence of asymmetry and small-study effects, and, therefore, no strong evidence of publication bias (Figure 5B and Suppl Figure S2B).

Furthermore, in the observational studies, some subgroup analyses were performed to check the possible interference of some parameters in modifying the efficacy of TCZ therapy on COVID-19 mortality.

**Figure 6:**
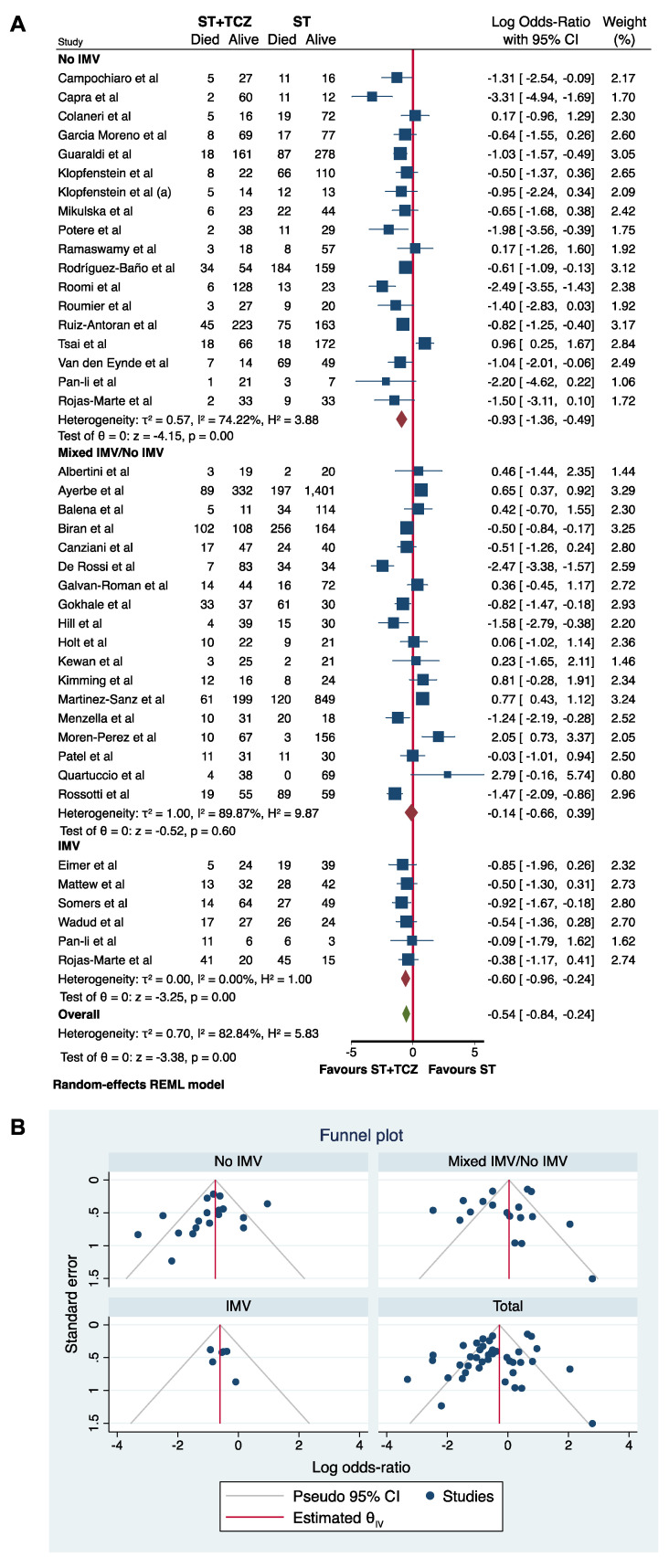
(**A**) Forest plot and (**B**) funnel plot of the subgroup analysis on the observational studies stratified by IMV use.

Firstly, a subgroup analysis was performed using IMV at the initiation of TCZ treatment as a stratifying variable (Figure 6). The meta-analysis of all 40 studies divided by IMV showed a statistically significant higher survival rate in patients treated with TCZ in the No-IMV and IMV groups (Log odds ratio = −0.93; 95% CI: −1.36 −0.49; *p* < 0.001 and Log odds ratio = −0.60; 95% CI: −0.96 −0.24; *p* < 0.001, respectively), but not in the mixed No-IMV/IMV group (Log odds ratio = −0.14; 95% CI: −0.66 0.39; *p* = 0.60), suggesting the importance of the patient’s assessment in the choice of the TCZ therapy, and in the data interpretation (Figure 6A). Rojas-Marte’s [57] and Pan-Li’s [51] studies provided the data divided for No-IMV and IMV use; accordingly, the subgroup analysis was performed, taking into account this stratification. In Figure 6B, the funnel plot is shown.

**Figure 7:**
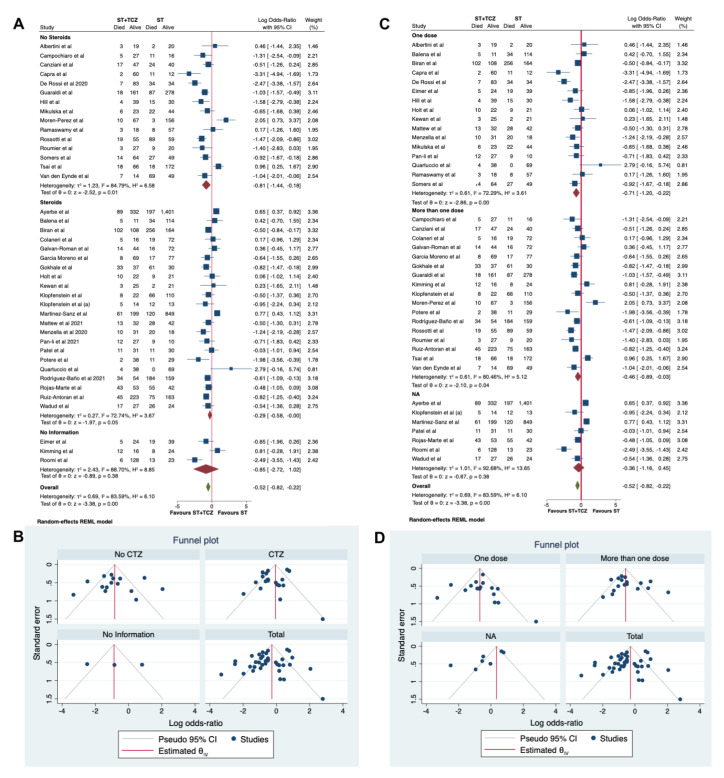
(**A**) Forest plot and (**B**) funnel plot of the subgroup analysis on the observational studies stratified by use of steroids. (**C**) Forest plot and (**D**) funnel plot of the subgroup analysis on the observational studies stratified by number of TCZ dose received.

Moreover, a second subgroup analysis was performed, taking into account the use of steroids in the ST (Figure 7A,B). A statistically significant higher survival rate was found in patients treated with TCZ in the No-Steroids group (Log odds ratio = −0.81; 95% CI: −1.44, −0.18; *p* < 0.001), but not in the group without information (Log odds ratio = −0.85; 95% CI: −2.72 1.02; *p* = 0.38). Only a trend was found in the group using steroids (Log odds ratio = −0.29; 95% CI: −0.58 −0.00; *p* = 0.05; Figure 7A). This last result could help to explain which of the well-known effects of steroids on the immune systems could have interfered with the TCZ effect. In Figure 7B, the funnel plot is shown.

In a third subgroup analysis, the different dosage regimen of TCZ was considered as a stratifying variable (one dose vs. more than one dose, Figure 7C,D). A statistically significant higher survival rate was found in patients treated with TCZ in both the groups treated with one or more doses (Log odds ratio = −0.71; 95% CI: −1.20 −0.22; *p* < 0.001 and Log odds ratio = −0.46; 95% CI: −089 −0.003; *p* = 0.04, respectively), but not in the group without information (Log odds ratio = −0.36; 95% CI: −1.16 0.45; *p* = 0.38, Figure 7C). In Figure 7D, the funnel plot is shown.

### 3.7. Meta-Regression Analyses

Since the differences in the mortality rate between patients treated with ST+TCZ and ST alone were characterized by a high heterogeneity (I^2^ ≥ 50%, *p* < 0.05), in the observational studies we ran a meta-regression analysis to seek potential moderators, such as age and percentage of the female population. Both these parameters did not affect the findings (age: beta −0.005; 95% CI: −0.068 to 0.058; *p* = 0.868; percentage of female: beta = 0.034; 95% CI: −0.004 to 0.072; *p*= 0.0780), confirming the efficacy of the treatment with ST + TCZ in reducing mortality in respect to ST alone. In addition, to exclude the possibility that other factors (i.e., the use of IMV (yes vs. mixed/no IMV), use of steroids (yes/no), the use of one or more TCZ doses, the timing of TCZ administration, and the mode of TCZ administration (IV vs. SC or IV/SC)) can affect the effect size, we performed other meta-regressions (Table S1A–I). By adding to age and percentage of female population, the use of IMV (Table S1C), one TCZ dose (Table S1D), or the use of steroids (Table S1G,H), as moderators, the finding was confirmed. The result was the same, whether adding them individually or in a cumulative analysis (Table S1). The timing of TCZ administration also did not affect the result (Table S1E), likely because this parameter, in some studies, represented the time from hospitalization to TCZ administration, in others the time from symptom onset to TCZ administration. On the contrary, the intravenous mode of TCZ administration was found to moderate the results (beta = −0.574; 95% CI: −0.910 to 0.239; *p* = 0.001; Table S1F). This effect was also confirmed when all the variables were introduced in a cumulative model (Table S1I), where the only parameter modulating the results was the mode of administration (beta = −0.820; 95% CI: −1.355 to −0.285; *p* = 0.003; Table S1I). However, the small number (n = 5) of studies adopting another mode of administration could explain this finding.

Similarly, we ran a meta-regression analysis in the RCTs to seek potential moderators, such as age, percentage of the female population, and use of IMV. These parameters did not affect the findings (age: beta −0.086; 95% CI: −0.188 to 0.016; *p* = 0.099; percentage of female: beta= −0.033; 95% CI: −0.155 to 0.088; *p*= 0.590; use of IMV: beta −0.194; 95% CI: −1.280 to 0.890; *p* = 0.725), confirming that the RCTs did not achieve any conclusive results on the efficacy of TCZ to reduce COVID-19 mortality.

## 4. Discussion

The present meta-analysis demonstrates that treatment with TCZ *plus* ST compared with ST alone reduces the mortality rate in COVID-19 patients.

This finding is consistent with the results of previous meta-analyses reporting a reduction in mortality in COVID-19 patients treated with TCZ added to ST in comparison with those treated with ST alone [73,74,75,76,77,78].

Recently, Chen et al. found that, overall, TCZ decreased the relative risk of death in COVID-19 patients, but this finding was not confirmed by analyzing randomized trials or studies with a concurrent control cohort. However, these authors included only three RCTs and the heterogeneity of the studies with a concurrent control cohort was high [79]. 

In the present study, since there was high heterogeneity in the observational studies, we performed a sensitivity analysis, the effect of TCZ was confirmed in 33/40 observational studies, involving 1945 subjects with ST + TCZ and 3187 with ST alone (I^2^ = 13.72%, *p* < 0.001). This finding allowed us to draw a real-world picture related to the beneficial effect of TCZ on COVID-19 mortality.

Considering the extreme heterogeneity found in the primary analysis of 40 observational studies, mainly related to differences in the patients’ clinical characteristics, we performed a subgroup analysis based on whether the patients were using IMV at the initiation of the TCZ treatment. 

A statistically significant mortality reduction associated with TCZ was found in the no-IMV and in the IMV groups. Conversely, the group of studies enrolling a No-IMV/IMV population did not reveal a significant association between TCZ and mortality rate. Of note, Patel et al. [52] enrolled patients with severe and critical illness and only found a decreasing trend in mortality in the group of severe TCZ treated patients compared with the controls (14.2% vs. 28.6%). The authors reported an average timing of 12 days from symptom onset to TCZ initiation, which is likely a delayed time to allow benefits, especially in critically ill patients [52]. Similarly, Galvan-Roman et al. reported a median time of 11 days between symptom onset and the drug administration (IQR 8–12.5). The authors showed that patients treated after an average time of 6 days (referred to as early TCZ) showed an improvement in inflammatory parameters [36]. The timing of the TCZ administration is an important variable, as it is highlighted in several studies included in the present meta-analysis, and by the investigators of the Study of the Treatment and Outcomes in Critically Ill Patients With COVID-19 (STOP-COVID tocilizumab study) [80]. Moreover, in the studies by Kimmig et al. and Quartuccio et al., the authors highlighted that TCZ was administered in a stage of the disease too advanced to be effective [43,54].

In addition, Rojas-Marte et al. [57], who enrolled a mixed No-IMV/IMV population, also found a statistically significant decreased mortality only when the patients using IMV were excluded. Therefore, we considered the patients’ data separately, as shown in our subgroup analysis.

Overall, a significant effect in reducing the death rates was found by analyzing the studies [35,47,51,57,62,65] evaluating critically ill patients receiving IMV when TCZ was administered. 

Critically ill patients were also examined in the aforementioned STOP-COVID study performed by Gupta et al. [80]. This is a large retrospective study, structured to emulate a hypothetical target trial in which the time to death is the main endpoint. The subjects administered with TCZ after the first 2 days were included in the non-TCZ-treated group as well as the patients who received ST alone. The risk of in-hospital mortality was lower in the subjects treated with TCZ in the first 2 days of their ICU stay, compared with those whose treatment was delayed. The beneficial effect of TCZ on mortality was particularly pronounced in patients admitted to the ICU within 3 days of the onset of symptoms [80]. The study design represents strength, as it underlines the need for early administration of TCZ in patients using IMV, but the lack of a comparative untreated group prompted the exclusion of this study from our meta-analysis.

It is important to remark that in both the studies by Somers et al. [62] and Gupta et al. [80] the patients treated with TCZ were younger as compared to those of the control groups. However, our meta-regression analysis did not reveal any influence of the age variable.

According to previous studies [77,78,79], the results of the meta-analysis conducted on RCTs showed a lack of significant association between TCZ treatment and decreased mortality rate, whereas a low heterogeneity was found (I^2^ = 31.18, Figure 2A).

As discussed by Parr [15], some of these RCTs are not adequate to clarify whether TCZ is useful in COVID-19 patients. Two of them [67,70] have a small sample size, and RCT-TCZ-COVID-19 [70] reported an unrealistic 2.4% overall mortality. A recent case fatality rate of 13.2% was reported in Italy [81].

Recently, as part of the Randomized Evaluation of COVID-19 Therapy (RECOVERY), the results on the safety and efficacy of TCZ in COVID-19 patients demonstrated that TCZ significantly reduced the mortality rate in patients who received ST+TCZ when compared with those administered with ST alone (29% vs. 33%, *p* = 0.007) [82].

An important issue is the concomitant administration of steroids that could influence the effect of TCZ. In a study by Gupta et al. [80], the TCZ group was more likely to be administered with steroids that have a proven efficacy in reducing mortality among patients who receive supportive oxygen therapy. Our subgroup analysis demonstrated that the administration of steroids within ST could slightly diminish the efficacy of TCZ in reducing the mortality rate. This is not a surprising result since steroids could lower the levels of cytokines, including IL-6, which is the TCZ molecular target. Indeed, individually, several studies suggest that the TCZ benefit is unrelated to steroid use. Mikulska et al. [49] found that early treatment with TCZ, methylprednisolone, or both reduces the mortality rate. Somers et al. [62], who analyzed a well-balanced-patient population stratified by concomitant treatments including steroids, also found a statistically significant reduction in mortality in the TCZ group. Kewan et al. [42] reported that treatment with TCZ improved clinical symptoms faster than ST alone, regardless of the concomitant use of steroids. Moreover, in a study by Ramaswamy et al. [55], there was no difference in the mortality rate in the two groups, although the patients administered with TCZ were more often treated with steroids. A further sub-group analysis revealed no potential interference of dosage regimen on the TCZ effect. 

Another concern regards the timing of TCZ administration. Some studies considered the timing from the onset of symptoms and others from hospital admission to TCZ initiation. The absence of a univocal evaluation does not allow an assessment of the influence of this crucial aspect on mortality. 

Among the RCTs, only BACC [66], COVACTA [68], and EMPACTA [69] are double-blind placebo RCTs. The first [66] enrolled only moderately ill patients and concluded that TCZ was ineffective in preventing IMV use and death. The investigators of STOP-COVID [83] have highlighted that the results of this RCT should not be extrapolated to severe patients, especially those with a critical illness. Actually, the patients in the STOP-COVID study (100% admitted in ICU and 60.6% requiring IMV) profoundly differ from those in the BACC study (4% of patients in an ICU and 0% requiring IMV) [83].

Conversely, COVACTA (enrolling severe and critical patients) and EMPACTA (enrolling only severe patients) demonstrated the usefulness of TCZ in shortening the length of stay but failed to find an impact in reducing the mortality rate [68,69].

Notably, as shown in our subgroup analysis, in the observational studies enrolling a mixed population of patients (No-IMV/IMV) there is no statistically significant difference between the ST+TCZ and ST groups. This finding is similar to the result of the meta-analysis on the RCTs, the majority of them enrolled a mixed population, and in our study the subgroup analysis by IMV on RCTs did not achieve any conclusive result (Figure 4).

## 5. Conclusions

Overall, the present meta-analysis demonstrates that TCZ reduces the COVID-19 mortality rate. This finding is evident considering the observational studies but not the RCTs. 

However, RECOVERY found a statistically significant reduction in the mortality rate associated with the use of TCZ. It is noteworthy that the other RCTs included in our meta-analysis, considered altogether, enrolled less than half of the TCZ-treated patients enrolled in this RCT.

Nonetheless, interpreting the results of both observational studies and RCTs is arduous due to the heterogeneity in COVID-19 severity of the enrolled patients. Moreover, different study designs and lack of important data, such as the timing of TCZ administration from symptoms onset, hamper a conclusive evaluation of the TCZ impact on COVID-19 mortality rate.

In the near future, it will be very important to take into account the stage of disease and patients’ characteristics, following a personalized therapeutic approach.

## Figures and Tables

**Table 1:** PICO format used to focus the research question.

Condition	Definition
Population	COVID-19 patients admitted in hospital
Intervention	Patients treated with Tocilizumab plus Standard Therapy
Comparator	Patients treated with Standard Therapy alone
Outcome	Mortality

**Table 2:** Characteristics of the observational studies introduced in the meta-analysis.

Source	Country	Primary Outcome	Total Pts (n)	Mean Age	Sex, (Male %)	Oxygen Support	TCZ Administer Route and Dosage	Time from Symptoms Onset to TCZ Start (days)	Time from Hospital Admission to TCZ Start (days)	Death (%) (*p* Value)
Albertini [26]	Europe	Efficacy of TCZ on respiratory clinical conditions	44 TCZ: 22 ST: 22	65	70.45	No-IMV/IMV	8 mg/kg	10	NA	TCZ: 13.6 ST: 9 (*p* = NA)
Ayerbe [27]	Europe	Mortality with Heparin therapy	2019 TCZ: 421 ST: 1598	67.57	61.268	No-IMV/IMV	NA	NA	NA	TCZ: 21.14 ST: 12.33 (*p* = NA)
Balena [28]	Europe	Crude mortality and AdE	164 TCZ:16 ST:148	77.5	48	No-IMV/IMV	8 mg/kg i.v.	NA	NA	TCZ: 31 ST:23 (*p* = 0.074)
Biran [29]	America	Mortality in patients requiring ICU	630 TCZ:210 ST:420	65	58.413	No-IMV/IMV	8 mg/kg i.v.	7	NA	TCZ: 49 ST: 61 **(*p* = 0.0040)**
Campochiaro [30]	Europe	Clinical improvement and overall survival	59 TCZ:32 ST:27	62	86.154	No- IMV	400 mg i.v., repeated after 24 h (n = 9)	11	NA	TCZ: 16 ST: 33 (*p* = 0.15)
Canziani [31]	Europe	Mortality	128 TCZ:64 ST:64	63.5	73	No-IMV/IMV	8 mg/kg i.v., repeated after 24 h (n = 61)	13	NA	TCZ: 27 ST: 38 (*p* = 0.185)
Capra [32]	Europe	Mortality	85 TCZ:62 ST:23	66.5	75	No- IMV	400 mg i.v. 324 mg s.c.	NA	4	TCZ: 3.22 ST: 47.8 **(*p* = 0.004)**
Colaneri [33]	Europe	ICU admission and 7-day mortality rate	112 TCZ:21 ST:91	63.03	73.215	No- IMV	8 mg/kg i.v. and repeated after 12 h	NA	NA	TCZ: 24 ST: 21 (*p* = 0.84)
De Rossi [34]	Europe	Survival rate	158 TCZ:90 ST:68	66.95	71.52	No- IMV/IMV	400 mg i.v. or 324 mg s.c.	NA	9	TCZ: 7.7 ST: 50 **(*p* < 0.001)**
Eimer [35]	Europe	30-day death after admission to ICU	87 TCZ:29 ST:58	56.5	84	IMV	8 mg/kg i.v.	11	NA	TCZ: 17.2 ST: 32.8 (*p* = 0.20)
Galvan-Romàn [36]	Europe	Need for IMV, mortality	146 TCZ: 58 ST: 88	63	66	No-IMV/IMV	8 mg/kg (max 800 mg), repeated after 12 h	NA	NA	TCZ: 24 ST: 18 (*p* = NA)
Garcia [37]	Europe	ICU admission and/or death	171 TCZ: 77 ST: 94	61	65.498	No- IMV	400 mg/24 h iv (Pts ≤ 75 kg) 600 mg/24 h iv (Pts > 75 kg) with the possibility to repeat the dose every 12 h up to 3 doses.	NA	6.5	TCZ: 10.3 ST: 18 (*p* = 0.156)
Gokhale [38]	Asia	Overall survival	161 TCZ: 70 ST: 91	53.5	62.11	No-IMV/IMV	400 mg/die i.v. and repeated after 24 h (n = 9)	NA	12	TCZ: 47 ST: 67 **(*p* = 0.011)**
Guaraldi [39]	Europe	IMV requirement and/or death	544 TCZ: 179 ST: 365	67	66	No- IMV	8 mg/kg i.v., repeated after 12 h, or 324 mg s.c.	NA	7	TCZ: 7 ST: 20 **(*p* < 0.001)**
Hill [40]	America	Clinical improvement	88 TCZ: 43 ST: 45	NA	69	No-IMV/IMV	400 mg i.v.	NA	2	TCZ: 21 ST: 33 (*p* = 0.26)
Holt [41]	America	Survival time and mortality	62 TCZ: 32 ST: 30	68.5	70.97	No-IMV/IMV	400 mg i.v.	NA	2	TCZ: 31.25 ST: 30 (*p* = 0.36)
Kewan [42]	North America	Median LOS, ICU LOS, duration of IMV, mortality.	51 TCZ: 28 ST: 23	66	61	No-IMV/IMV	400 mg i.v.	NA	2	TCZ: 11 ST: 9 (*p* > 0.99)
Kimmig [43]	North America	Infection and clinical outcomes (discharged, died)	60 TCZ:28 ST: 32	63.15	55.856	No-IMV/IMV	400 mg i.v. with possible redosing	NA	NA	TCZ: 35.2 ST: 19.3 **(*p* = 0.020)**
Klopfenstein [44]	Europe	IMV requirement and/or death	206 TCZ: 30 ST;176	73.75	60.7	No- IMV	8 mg/kg i.v. (1 or 2 doses)	12	NA	TCZ: 26.7 ST: 37.5 (*p* = 0.253)
Klopfenstein [45]	Europe	ICU admission and/or death	44 TCZ: 19 ST: 25	74.95	NA	No- IMV	NA	13	NA	TCZ: 25 ST: 48 (*p* = 0.066)
Martinez-Sanz [46]	Europe	Time to death	1229 TCZ: 260 ST: 969	65.5	72.246	No-IMV/IMV	NA	NA	4	TCZ: 23 ST: 12 **(*p* < 0.001)**
Matthew [47]	America	Overall mortality 30 days from the date of intubation	115 TCZ: 45 ST: 70	58.4	69.566	IMV	400 mg i.v.	NA	2.5	TCZ: 29 ST: 40 (*p* = 0.23)
Menzella [48]	Europe	In-hospital mortality rate	79 TCZ: 41 ST: 38	66.5	70.89	No-IMV/IMV	8 mg/kg i.v (max 800 mg) or 162 mg s.c	NA	NA	TCZ: 24 ST: 53 **(*p* = 0.01)**
Mikulska [49]	Europe	Failure-free survival and overall survival	95 TCZ: 29 ST: 66	69	67.35	No- IMV	8 mg/kg i.v. 162 mg s.c.	NA	7	TCZ: 14.2 ST: 28.1 (*p* = NA)
Moreno-Pérez [50]	Europe	Death, LOS	236 TCZ: 77 ST: 159	59.5	59.746	No-IMV/IMV	600 mg i.v., with second or third dose (400 mg i.v.)	10	NA	TCZ: 12.9 ST: 1.9 **(*p* = 0.002)**
Pan-Li [51]	Asia	Improvement and death	58 TCZ: 39 ST: 19	73.9	63.8	No- IMV/IMV	4–8 mg/kg (max dose of 800 mg)	NA	NA	TCZ: 30.8 ST: 47.3 (*p* = NA)
Patel [52]	America	Clinical outcomes and survival	83 TCZ: 42 ST: 41	67.5	50.603	No-IMV/IMV	NA	NA	4	TCZ: 21.4 ST: 26.8 in severe Pts TCZ: 14.2 ST: 28.6 (*p* = NA)
Potere [53]	Europe	Overall survival and survival-free of IMV	80 TCZ: 40 ST: 40	55.25	65	No- IMV	324 mg s.c. (bid)	NA	5	TCZ: 5 ST: 27.5 **(*p* = 0.006)**
Quartuccio [54]	Europe	Optimal patient selection to be treated with TCZ	111 TCZ: 42 ST: 69	58.3	69.4	No-IMV/IMV	8 mg/kg i.v.	8.4	NA	TCZ: 9.5 ST: 0 (*p* = NA)
Ramaswamy [55]	North America	Mortality	86 TCZ: 21 ST: 65	63.7	57	No- IMV	400 mg i.v. 8 mg/kg i.v.	NA	NA	TCZ: 14 ST: 12 (*p* = 0.81)
Rodríguez-Bano [56]	Europe	Intubation or death	432 TCZ: 88 ST: 343	67.5	77.702	No- IMV	400–600 mg i.v. with second or third dose	10	NA	TCZ: 2.3 ST: 11.9 **(*p* = 0.004)**
Rojas-Marte [57]	North America	Mortality	193 TCZ = 96 ST = 97	60.4	71	No-IMV/IMV	NA	NA	NA	TCZ: 52 ST: 62 (*p* = 0.09) excluding intubated TCZ: 6 ST: 27 **(*p* = 0.024)**
Roomi [58]	America	Clinical effectiveness of HCQ and TCZ	170 TCZ: 134 ST: 36	61.8	48.83	No- IMV	NA	NA	NA	TCZ: 4.5 ST: 36 (*p* = 0.44)
Rossotti [59]	Europe	Overall survival and hospital discharge	222 TCZ: 74 ST: 148	59	81.532	No-IMV/IMV	8 mg/kg i.v. (max dose of 800 mg) with possible second dose	NA	NA	TCZ: 25.7 ST: 60.1 **(*p* = 0.035)**
Roumier [60]	Europe	IMV requirement and death	59 TCZ: 30 ST: 29	65	80	No- IMV	8 mg/kg i.v. (renewable once)	14	NA	TCZ: 17.2 ST: 18.7 (*p* = 0.837) unadjusted TCZ: 10 ST: 31 **(*p* = 0.41)**
Ruiz-Antora’n [61]	Europe	Mortality	506 TCZ: 268 ST: 238	68	64.03	No- IMV	600 mg (3 doses n = 22, 2 doses n = 92, 1 dose n = 154)	11	NA	TCZ: 16,8 ST: 31,5 (28days/ death) **(*p* = 0.001)**
Somers [62]	North America	Survival probability after intubation	154 TCZ = 78 ST = 76	58	66	IMV	8 mg/kg i.v. (max 800 mg)	NA	3.9	18% in TCZ 36% in ST (28days/ death) **(*p* = 0.01)**
Tsai [63]	America	Mortality	274 TCZ: 84 ST: 190	63	61.4	No- IMV	400 mg i.v. (n = 53) 600 mg i.v. (n = 3) 800 mg i.v (n = 10) (second dose n = 4)	NA	NA	TCZ: 21.4 ST: 9.4 (*p* = NA)
Van den Eynde [64]	Europe	Mortality	139 TCZ = 21 ST = 118	73.2	66.91	No- IMV	400 mg or 600 mg (once or twice daily)	NA	NA	TCZ: 33,3 ST: 58,4 **(*p* < 0.001)**
Wadud [65]	North America	LOS, days on ventilator, in-hospital and ICU, mortality.	94 TCZ: 44 ST: 50	55.5	NA	IMV	NA	NA	NA	3TCZ: 8.64 ST: 52 **(*p* < 0.001)**

Pts, Patients; TCZ, Tocilizumab; ST, Standard Therapy; AdE, adverse events; HCQ, hydroxychloroquine; LOS, length of stay; IMV, Invasive Mechanical Ventilation; NA, Not Available. In the Table the *p*-values statistically significant are reported in bold.

**Table 3:** Characteristics of the RCTs introduced in the meta-analysis.

Source	Country	Primary Outcome	Total Pts (n)	Mean Age	Sex, (Male %)	Oxygen Support	TCZ Administration Route and Dosage	Time from Symptoms Onset to TCZ Initiation (days)	Time from Hospital Admission to TCZ Initiation (days)	Death (%) *(p* Value*)*
BACC Stone [66]	America	Intubation or death	242 TCZ:161 ST:81	59.8	58.05	No-IMV	8 mg/kg i.v.	NA	9	TCZ: 5.6 ST: 4.9 *(p* = 0.81)
CORIMUNO-19 Hermine [67]	Europe	Death or respiratory support or IMV.	130 TCZ:63 ST:66	63.5	NA	No-IMV	8 mg/kg i.v.	NA	10	TCZ: 11.1 ST: 9 (day 14) (*p* = NA)
COVACTA Rosas [68]	America, Europe	Clinical Status	438 TCZ: 294 ST: 144	60.75	69.863	No-IMV/IMV	8 mg/kg i.v.	12	NA	TCZ: 19.7 ST: 19.4 (*p* = 0.941)
EMPACTA Salama [69]	America, Africa	IMV or death by day 28	377 TCZ:249 ST:128	55.9	NA	No-IMV	8 mg/kg i.v.	NA	8	TCZ: 10.4 ST: 8.6 (day 28) (*p* = NA)
RCT-TCZ-COVID-19 Salvarani [70]	Europe	Clinical worsening within 14 days since randomization	123 TCZ:60 ST:63	60	61.1	No-IMV	8 mg/kg i.v., repeated after 12h	NA	7	TCZ 1.7 ST 1,6 (day 14) (*p* = NA)
RECOVERY Recovery Group [71]	Europe	All-cause mortality within 28 days after randomization	4116 TCZ:2022 ST:2094	63.6	67.4	No-IMV/ IMV	400 mg i.v. 600 mg i.v. 800 mg i.v	9	NA	TCZ: 29 ST: 33 **(*p*= 0.007)**
TOCIBRAS Veiga [72]	America	Clinical status at 15 days	129 TCZ:65 ST:64	57.4	NA	No-IMV/ IMV	8 mg/kg i.v.	10	NA	TCZ: 17 ST: 3 (*p* = NA)

Pts, Patients; TCZ, Tocilizumab; ST, Standard Therapy; IMV, Invasive Mechanical Ventilation; NA, Not Available. In the Table the *p*-values statistically significant are reported in bold.

## Data Availability

The data that support the findings of this study are openly available. These data were derived from the resources available in the public domain described in Table 2.

## Supplementary Materials

The following are available online at https://www.mdpi.com/article/10.3390/jpm11070628/s1, Figure S1. The Cochrane Risk of Bias tool (A) with GRADE Analysis (B) for the quality assessment of the RCTs; Figure S2. The Newcastle-Ottawa Scale (A) with GRADE Analysis (B) for the quality assessment of the Observational studies; Figure S3. (A) Forest plot of the sensitivity analysis performed with a Fixed-effects Mantel-Haenszel model; Table S1: Metaregression analyses performed to evaluate the possible influence of other factors on the effect size: (A) age; (B) age and female percentage; (C) age, female percentage and IMV use (yes/no); (D) age, female percentage, dose (one/more than one); (E) age, female percentage, timing of TCZ administration; (F) age, female percentage, mode of TCZ administration (Intravenous/Other mode); (G) corticosteroid use; (H) age, female percentage, corticosteroid use; (I) cumulative metaregression analysis with all the considered factors.
