# Supplementary material for: Effect of Tocilizumab in Reducing the Mortality Rate in COVID-19 Patients: A Systematic Review with Meta-Analysis
Source: J Pers Med. 2021 Jul 1;11(7):628. doi: 10.3390/jpm11070628 (PMC8307114; doi:10.3390/jpm11070628)
Supplement: Supplementary file 1 [file jpm-11-00628-s001.zip › jpm-1267049-supplementary.pdf]

A

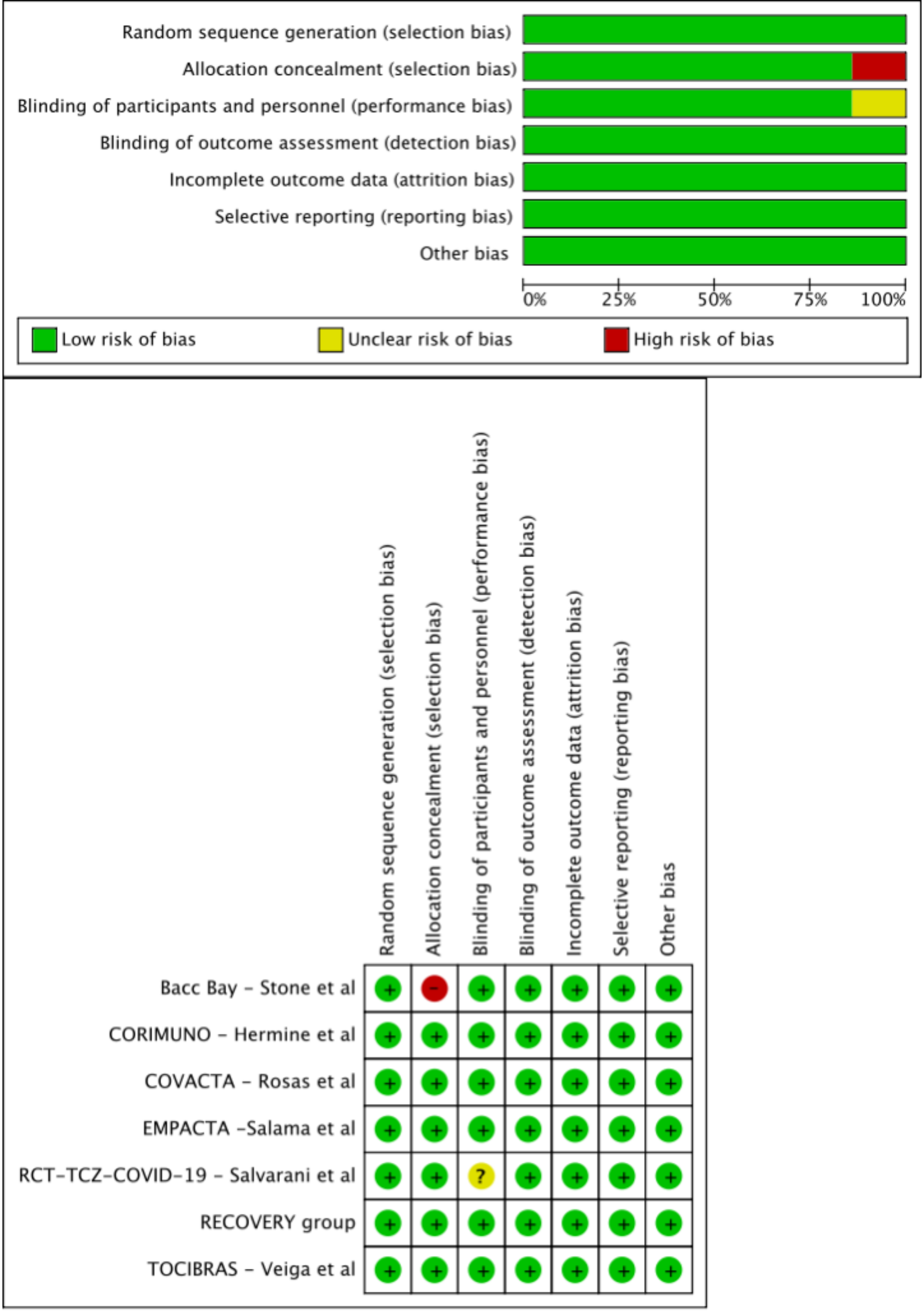

B

Certainty assessment							Summary of findings				
							Study event rates (%)			Anticipated absolute effects	
Participants (studies) Follow up	Risk of bias	Inconsistency	Indirectness	Imprecision	Publication bias	Overall certainty of evidence	With ST	With ST+TCZ	Relative effect (95% CI)	Risk with ST	Risk difference with ST+TCZ
**Mortality (follow up: mean 28 days)**											
5555 (7 RCTs)	not serious	not serious	not serious	not serious	none	⊕⊕⊕⊕ HIGH	749/2641 (28.4%)	711/2914 (24.4%)	**OR 0.89** (0.79 to 1.00)	284 per 1.000	**23 fewer per 1.000** (from 45 fewer to 0 fewer)

CI: Confidence interval; **OR:** Odds ratio

Summary of findings:

TCZ+ST compared to ST for COVID-19

Patient or population: COVID-19
Setting: hospital
Intervention: TCZ+ST
Comparison: ST

Outcome № of participants (studies)	Relative effect (95% CI)	Anticipated absolute effects (95% CI)			Certainty	What happens
				Difference		
Mortality follow up: mean 28 days № of participants: 5555 (7 RCTs)	**OR 0.89** (0.79 to 1.00)	28.4%	**26.1%** (23.8 to 28.4)	**2.3% fewer** (4,5 fewer to 0 fewer)	⊕⊕⊕⊕ HIGH	

***The risk in the intervention group** (and its 95% confidence interval) is based on the assumed risk in the comparison group and the **relative effect** of the intervention (and its 95% CI).

CI: Confidence interval; **OR:** Odds ratio

GRADE Working Group grades of evidence
High certainty: We are very confident that the true effect lies close to that of the estimate of the effect
Moderate certainty: We are moderately confident in the effect estimate: The true effect is likely to be close to the estimate of the effect, but there is a possibility that it is substantially different
Low certainty: Our confidence in the effect estimate is limited: The true effect may be substantially different from the estimate of the effect
Very low certainty: We have very little confidence in the effect estimate: The true effect is likely to be substantially different from the estimate of effect

Suppl Fig. 1

A

	Albertini	Balena	Biran	Campochiaro	Canziani	Capra	Colaneri	Eimer	Galvan Roman	Garcia	Gokhale	Guaraldi	Hill	Holt	Kewan	Kimmig	Klopfenstein	Klopfenstein (a)	Mattew	Menzella	Mikulska	Pan Li	Patel	Potere	Quartuccio	Ramaswamy	Rodriguez Bano	Rojas-Marte	Roumier	Ruiz antoran	Somers	Van der Eynde	Wadud
**Selection**																																	
1) Is the case definition adequate?	*	*	*	*	--	*	*	--	*	--	--	*	*	*	--	*	*	*	*	*	*	*	--	--	*	*	*	*	--	*	*	*	*
2) Representativeness of the cases	*	*	*	*	*	*	*	*	*	*	*	*	*	*	*	*	*	*	*	*	*	*	*	*	*	*	*	*	*	*	*	*	*
3) Selection of controls	*	*	*	*	*	*	*	*	*	*	*	*	*	*	*	*	*	*	*	*	*	*	*	*	*	*	*	*	*	*	*	*	*
4) Definition of controls	*	*	*	*	*	*	*	*		*	*	*	*	*	*	*	*	*	*	*	*	*	*	*	*	*	*	*	*	*	*	*	*
**Comparability**																																	
1) Comparability of cases and controls on the basis of the design or analysis	*	**	**	**	**	*	*	*	*	*	*	**	**	**	*	**	**	*	*	**	**	**	*	*	*	**	**	*	**	*	*	*	**
**Exposure**																																	
1) Ascertainment of exposure	*	*	*	*	*	*	*	*	*	*	*	*	*	*	*	*	*	*	*	*	*	*	*	*	*	*	*	*	*	*	*	*	*
2) Same method of ascertainment for cases and controls	*	*	*	*	*	*	*	*	*	*	*	*	*	*	*	*	*	*	*	*	*	*	*	*	*	*	*	*	*	*	*	*	*
3) Non-response rate	*	*	*	*	*	*	*	*	*	*	*	*	*		*	*	*	*	*	*	*	*	*	*	*	*	*	*	*	*	*	*	*
**Total**	8	9	9	9	8	8	8	8	7	7	7	9	9	8	7	9	9	8	8	9	9	9	7	7	8	9	9	8	8	8	8	8	9

Summary of findings:

B

[ST+TCZ] compared to [ST] in [COVID-19 positive subjects]

Patient or population: [COVID-19 positive subjects]
Setting:
Intervention: [ST+TCZ]
Comparison: [ST]

Outcome<br>№ of participants<br>(studies)	Relative effect<br>(95% CI)	Anticipated absolute effects (95% CI)			Certainty	What happens
				Difference		
Mortality<br>№ of participants: 5125<br>(33 observational studies)	**OR 0.54**<br>(0.47 to 0.62)	38.4%	**25.2%**<br>(22.7 to 27.9)	**13.2% fewer**<br>(15,8 fewer to 10,5 fewer)	⊕⊕⊕◯<br>MODERATE	[ST+TCZ] likely reduces mortality.

***The risk in the intervention group** (and its 95% confidence interval) is based on the assumed risk in the comparison group and the **relative effect** of the intervention (and its 95% CI).

CI: Confidence interval; **OR:** Odds ratio

GRADE Working Group grades of evidence
High certainty: We are very confident that the true effect lies close to that of the estimate of the effect
Moderate certainty: We are moderately confident in the effect estimate: The true effect is likely to be close to the estimate of the effect, but there is a possibility that it is substantially different
Low certainty: Our confidence in the effect estimate is limited: The true effect may be substantially different from the estimate of the effect
Very low certainty: We have very little confidence in the effect estimate: The true effect is likely to be substantially different from the estimate of effect

Suppl Fig. 2

A

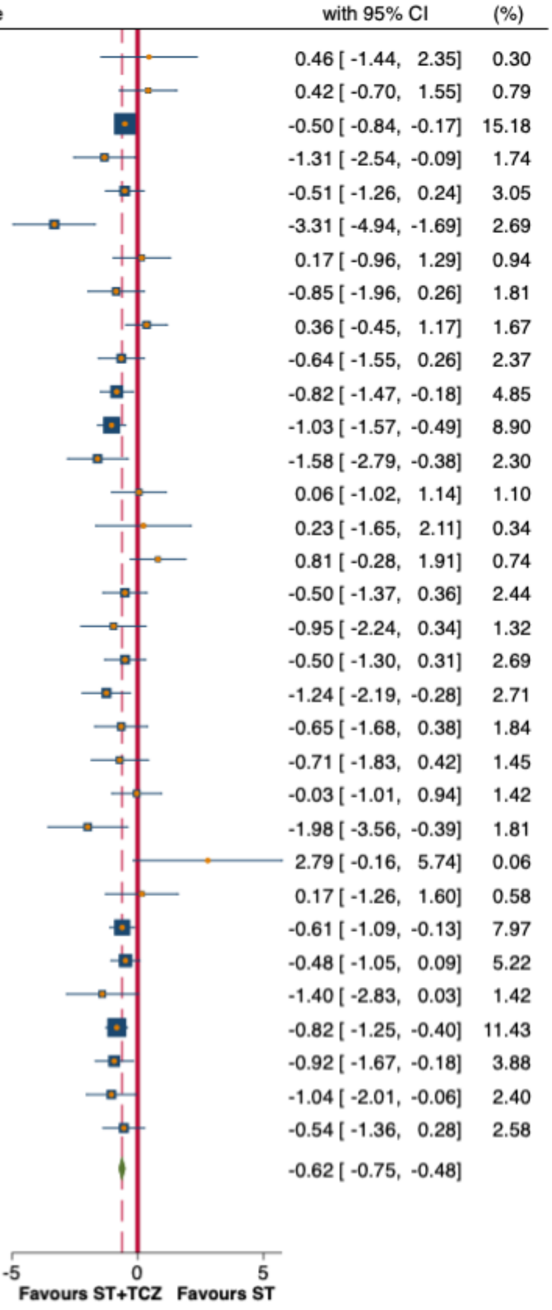

Study	ST+TCZ Died	ST+TCZ Alive	ST Died	ST Alive	Log Odds-Ratio with 95% CI	Weight (%)
Albertini et al	3	19	2	20	0.46 [-1.44, 2.35]	0.30
Balena et al	5	11	34	114	0.42 [-0.70, 1.55]	0.79
Biran et al	102	108	256	164	-0.50 [-0.84, -0.17]	15.18
Campochiaro et al	5	27	11	16	-1.31 [-2.54, -0.09]	1.74
Canziani et al	17	47	24	40	-0.51 [-1.26, 0.24]	3.05
Capra et al	2	60	11	12	-3.31 [-4.94, -1.69]	2.69
Colaneri et al	5	16	19	72	0.17 [-0.96, 1.29]	0.94
Eimer et al	5	24	19	39	-0.85 [-1.96, 0.26]	1.81
Galvan-Roman et al	14	44	16	72	0.36 [-0.45, 1.17]	1.67
Garcia Moreno et al	8	69	17	77	-0.64 [-1.55, 0.26]	2.37
Gokhale et al	33	37	61	30	-0.82 [-1.47, -0.18]	4.85
Guaraldi et al	18	161	87	278	-1.03 [-1.57, -0.49]	8.90
Hill et al	4	39	15	30	-1.58 [-2.79, -0.38]	2.30
Holt et al	10	22	9	21	0.06 [-1.02, 1.14]	1.10
Kewan et al	3	25	2	21	0.23 [-1.65, 2.11]	0.34
Kimming et al	12	16	8	24	0.81 [-0.28, 1.91]	0.74
Klopfenstein et al	8	22	66	110	-0.50 [-1.37, 0.36]	2.44
Klopfenstein et al (a)	5	14	12	13	-0.95 [-2.24, 0.34]	1.32
Mattew et al 2021	13	32	28	42	-0.50 [-1.30, 0.31]	2.69
Menzella et al 2020	10	31	20	18	-1.24 [-2.19, -0.28]	2.71
Mikulska et al	6	23	22	44	-0.65 [-1.68, 0.38]	1.84
Pan-li et al 2021	12	27	9	10	-0.71 [-1.83, 0.42]	1.45
Patel et al	11	31	11	30	-0.03 [-1.01, 0.94]	1.42
Potere et al	2	38	11	29	-1.98 [-3.56, -0.39]	1.81
Quartuccio et al	4	38	0	69	2.79 [-0.16, 5.74]	0.06
Ramaswamy et al	3	18	8	57	0.17 [-1.26, 1.60]	0.58
Rodríguez-Baño et al 2021	34	54	184	159	-0.61 [-1.09, -0.13]	7.97
Rojas-Marte et al	43	53	55	42	-0.48 [-1.05, 0.09]	5.22
Roumier et al	3	27	9	20	-1.40 [-2.83, 0.03]	1.42
Ruiz-Antoran et al	45	223	75	163	-0.82 [-1.25, -0.40]	11.43
Somers et al	14	64	27	49	-0.92 [-1.67, -0.18]	3.88
Van den Eynde et al	7	14	69	49	-1.04 [-2.01, -0.06]	2.40
Wadud et al	17	27	26	24	-0.54 [-1.36, 0.28]	2.58
**Overall**					-0.62 [-0.75, -0.48]	

Heterogeneity: $I^2 = 41.40\%$, $H^2 = 1.71$

Test of $\theta = 0$: $z = -8.88$, $p = 0.00$

Fixed-effects Mantel-Haenszel model

B

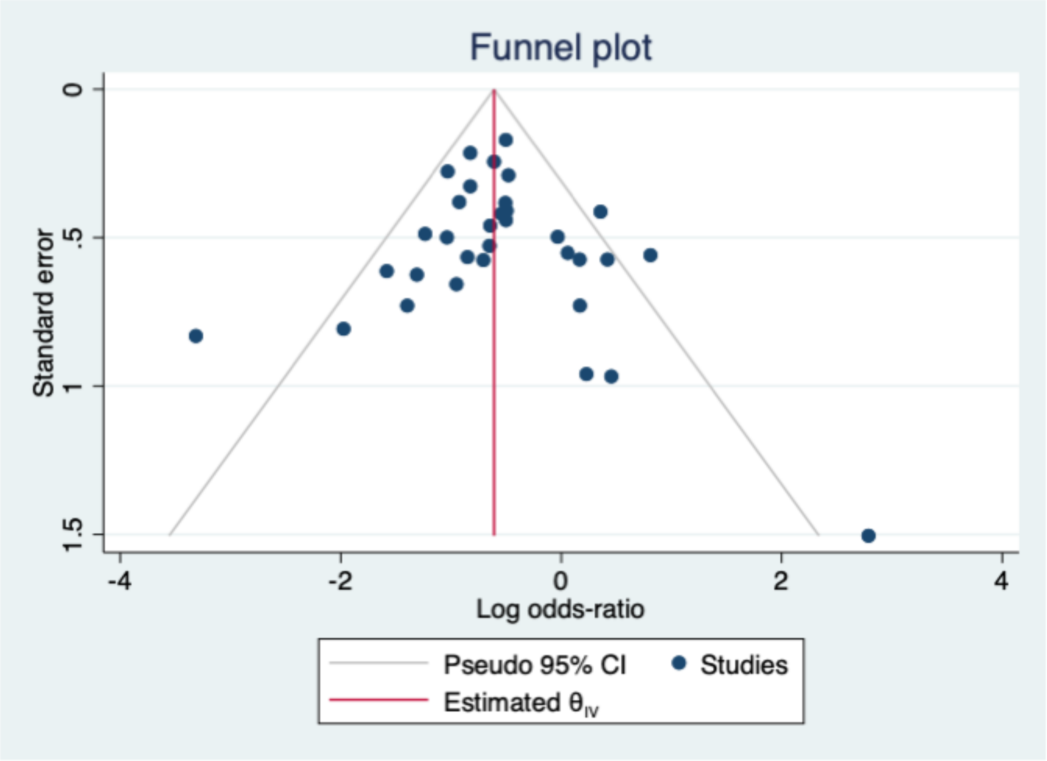

Suppl Fig. 3

A

Random-effects meta-regression
Method: REML

Number of obs =	39
Residual heterogeneity:	
tau2 =	.7111
I2 (%) =	84.17
H2 =	6.32
R-squared (%) =	0.00
Wald chi2(1) =	0.03
Prob > chi2 =	0.8544

_meta_es	Coef.	Std. Err.	z	P>|z|	[95% Conf.	Interval]
Age_total_mean	.0052704	.0287134	0.18	0.854	-.0510068	.0615477
_cons	-.8373497	1.858384	-0.45	0.652	-4.479715	2.805016

B

Random-effects meta-regression
Method: REML

Number of obs =	37
Residual heterogeneity:	
tau2 =	.7153
I2 (%) =	84.17
H2 =	6.32
R-squared (%) =	3.03
Wald chi2(2) =	3.20
Prob > chi2 =	0.2016

_meta_es	Coef.	Std. Err.	z	P>|z|	[95% Conf.	Interval]
Age_total_mean	-.0053736	.0324479	-0.17	0.868	-.0689703	.0582232
Female_tot_perc	.0338997	.0192361	1.76	0.078	-.0038023	.0716017
_cons	-1.266051	2.026761	-0.62	0.532	-5.23843	2.706328

C

Random-effects meta-regression
Method: REML

Number of obs =	35
Residual heterogeneity:	
tau2 =	.8224
I2 (%) =	85.96
H2 =	7.12
R-squared (%) =	0.00
Wald chi2(3) =	3.00
Prob > chi2 =	0.3912

_meta_es	Coef.	Std. Err.	z	P>|z|	[95% Conf.	Interval]
Age_total_mean	-.0055345	.0390578	-0.14	0.887	-.0820864	.0710174
Female_tot_perc	.0337998	.0204396	1.65	0.098	-.006261	.0738605
IMV_cat	-.1208417	.6808665	-0.18	0.859	-1.455315	1.213632
_cons	-1.238272	2.514126	-0.49	0.622	-6.165869	3.689325

D

Random-effects meta-regression
Method: REML

Number of obs =	37
Residual heterogeneity:	
tau2 =	.7255
I2 (%) =	83.23
H2 =	5.96
R-squared (%) =	1.65
Wald chi2(3) =	3.42
Prob > chi2 =	0.3317

_meta_es	Coef.	Std. Err.	z	P>|z|	[95% Conf.	Interval]
Age_total_mean	-.0033884	.0328899	-0.10	0.918	-.0678514	.0610746
Female_tot_perc	.031897	.0197744	1.61	0.107	-.00686	.0706541
Dose_cat	.1253835	.2550035	0.49	0.623	-.3744142	.6251812
_cons	-1.4229	2.06364	-0.69	0.491	-5.46756	2.62176

E

Random-effects meta-regression
Method: REML

Number of obs =	25
Residual heterogeneity:	
tau2 =	.7314
I2 (%) =	82.47
H2 =	5.70
R-squared (%) =	0.00
Wald chi2(3) =	2.41
Prob > chi2 =	0.4926

_meta_es	Coef.	Std. Err.	z	P>|z|	[95% Conf.	Interval]
Age_total_mean	-.0153946	.0415938	-0.37	0.711	-.096917	.0661278
Female_tot_perc	.038115	.0265789	1.43	0.152	-.0139787	.0902088
Timing_TCZ	-.0081562	.0587429	-0.14	0.890	-.1232902	.1069778
_cons	-.7211457	2.75289	-0.26	0.793	-6.116711	4.674419

F

Random-effects meta-regression
Method: REML

Number of obs =	32
Residual heterogeneity:	
tau2 =	.2786
I2 (%) =	60.53
H2 =	2.53
R-squared (%) =	53.74
Wald chi2(3) =	23.84
Prob > chi2 =	0.0000

_meta_es	Coef.	Std. Err.	z	P>|z|	[95% Conf.	Interval]
Age_total_mean	-.0114922	.0248185	-0.46	0.643	-.0601356	.0371511
Female_tot_perc	.0518066	.01654	3.13	0.002	.0193888	.0842244
Mode_injection	-.5746726	.171245	-3.36	0.001	-.9103066	-.2390386
_cons	-1.295176	1.533695	-0.84	0.398	-4.301164	1.710812

G

Random-effects meta-regression
Method: REML

Number of obs = 40
Residual heterogeneity:
tau2 = .6905
I2 (%) = 83.58
H2 = 6.09
R-squared (%) = 0.00
Wald chi2(1) = 0.80
Prob > chi2 = 0.3697

_meta_es	Coef.	Std. Err.	z	P>|z|	[95% Conf.	Interval]
Corticost_cat	.2347404	.2616668	0.90	0.370	-.2781172	.7475979
_cons	-.6887763	.2419221	-2.85	0.004	-1.162935	-.2146176

H

Random-effects meta-regression
Method: REML

Number of obs = 37
Residual heterogeneity:
tau2 = .7447
I2 (%) = 84.63
H2 = 6.51
R-squared (%) = 0.00
Wald chi2(3) = 3.17
Prob > chi2 = 0.3664

_meta_es	Coef.	Std. Err.	z	P>|z|	[95% Conf.	Interval]
Corticost_cat	.0621324	.291176	0.21	0.831	-.5085622	.632827
Age_total_mean	-.0044416	.033387	-0.13	0.894	-.0698789	.0609956
Female_tot_perc	.0325839	.0206067	1.58	0.114	-.0078045	.0729724
_cons	-1.326711	2.085264	-0.64	0.525	-5.413753	2.760331

Random-effects meta-regression | Number of obs = **23**
Method: REML | Residual heterogeneity:

tau2 =	**.4674**
I2 (%) =	**69.60**
H2 =	**3.29**
R-squared (%) =	**9.48**
Wald chi2(7) =	**13.25**
Prob > chi2 =	**0.0663**

_meta_es	Coef.	Std. Err.	z	P>|z|	[95% Conf.	Interval]
Age_total_mean	-.0143186	.042169	-0.34	0.734	-.0969684	.0683311
Female_tot_perc	.0381356	.0273911	1.39	0.164	-.01555	.0918211
Corticost_cat	-.2286777	.3767779	-0.61	0.544	-.9671488	.5097934
IMV_cat	-.5189578	.680771	-0.76	0.446	-1.853244	.8153287
Dose_cat	-.2485185	.4477769	-0.56	0.579	-1.126145	.629108
Timing_TCZ	-.0281051	.0657062	-0.43	0.669	-.1568869	.1006767
Mode_injection	-.8200898	.2729461	-3.00	0.003	-1.355054	-.2851253
_cons	-.0077662	3.090532	-0.00	0.998	-6.065097	6.049565

Suppl Table
